# Supplementary material for: Causal Relationship between Aging and Anorexia Nervosa: A White-Matter-Microstructure-Mediated Mendelian Randomization Analysis
Source: Biomedicines. 2024 Aug 16;12(8):1874. doi: 10.3390/biomedicines12081874 (PMC11351342; doi:10.3390/biomedicines12081874)
Supplement: Supplementary file 1 [file biomedicines-12-01874-s001.zip › Supplementary Figure/Figure S8/Leaveoneout plot of MR analysis for TL to ubm-b-1894.pdf]

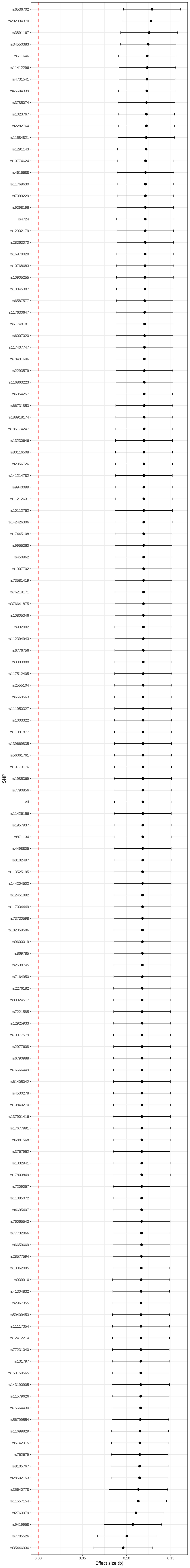
rs6536702
rs202034370
rs3891167
rs34550383
rs611646
rs11412296
rs4731541
rs45604339
rs3785074
rs1023767
rs2282764
rs11584821
rs1291143
rs10774624
rs4616688
rs11769630
rs7099229
rs9398196
rs4724
rs12932179
rs28363070
rs16978028
rs10768683
rs10905255
rs10845387
rs6587577
rs117630647
rs61748181
rs6007020
rs117407747
rs78491606
rs2293579
rs116863223
rs6054257
rs66731853
rs188918174
rs185174247
rs13230646
rs80116508
rs2056726
rs141214782
rs9940099
rs11212631
rs10112752
rs142426306
rs17445108
rs9955360
rs450962
rs1907702
rs73581419
rs76219171
rs376641875
rs10805346
rs932002
rs112394943
rs6776756
rs3093888
rs117512405
rs2555104
rs6669563
rs111950327
rs1003322
rs11991877
rs139669835
rs56061761
rs10773176
rs1985369
rs7790856
All
rs11426156
rs1957937
rs871134
rs4498805
rs8102497
rs113525195
rs144204502
rs12451892
rs117034449
rs73730598
rs182059586
rs9600019
rs869785
rs2538745
rs7164950
rs2276182
rs80324517
rs7221585
rs12925933
rs79977579
rs2977608
rs6790988
rs76666449
rs61405042
rs4530278
rs10840270
rs137901416
rs17677991
rs6881568
rs3767952
rs1332941
rs17803849
rs7209057
rs11085072
rs4695407
rs76065543
rs77732866
rs6659669
rs28577594
rs13062095
rs939916
rs41304832
rs2967355
rs59409453
rs11117354
rs12412214
rs77231040
rs131797
rs150150565
rs143190905
rs11579626
rs75664430
rs56799554
rs11699829
rs5742915
rs762679
rs8105767
rs28502153
rs35640778
rs11557154
rs2763979
rs9419958
rs7705526
rs35446936
SNP
0.00
0.05
0.10
0.15
Effect size (b)
